# Supplementary material for: Sensing Cells-Peptide Hydrogel Interaction In Situ via Scanning Ion Conductance Microscopy
Source: Cells. 2022 Dec 19;11(24):4137. doi: 10.3390/cells11244137 (PMC9776472; doi:10.3390/cells11244137)
Supplement: Supplementary file 1 [file cells-11-04137-s001.zip › cells-2089321-SI.pdf]

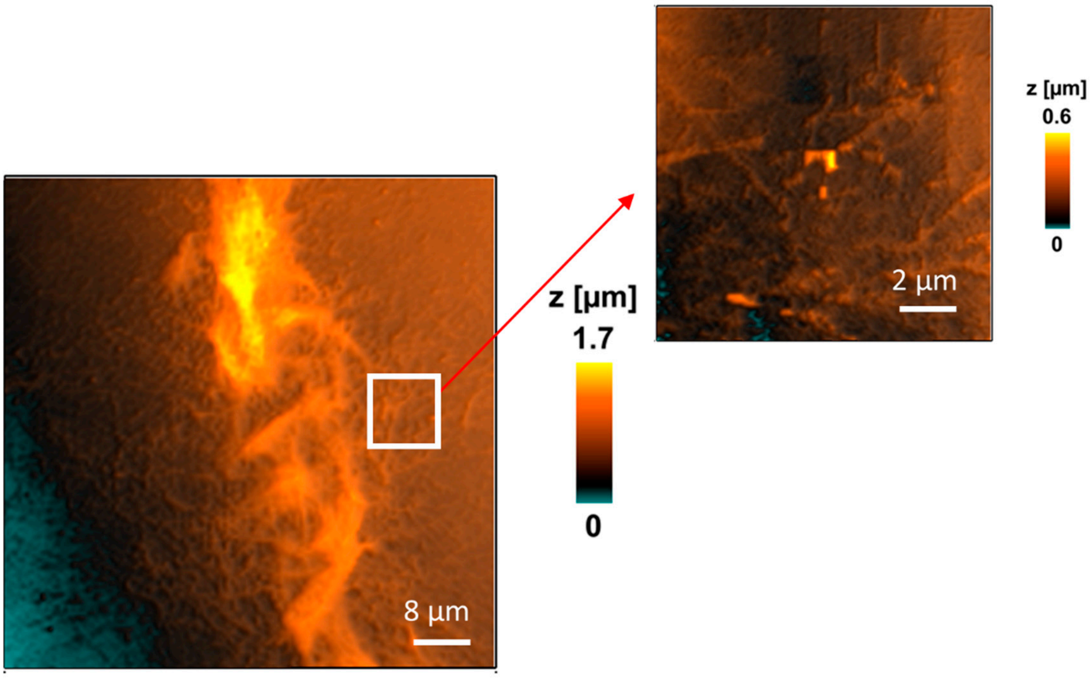

Figure S1. SICM topography image of Fmoc-FF hydrogel, that was formed, dried over the night after that the sample was immersed in the buffer and SICM images were obtained.

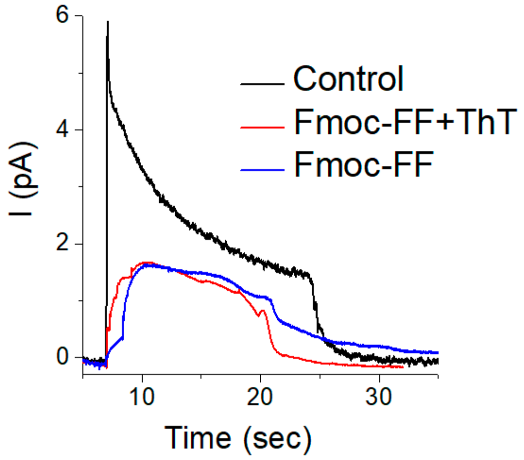

Figure S2. The dependences of current on time inside and outside the cells (MCF-7) for different substrates (1) Petri dish surface (control) (black line), (2) Fmoc-FF+ThT hydrogel (red line), (3) Fmoc-FF hydrogel (blue line).

Pt nanoelectrodes fabrication

For the fabrication of nanoelectrodes we used commercially available disk-shaped carbon nanoelectrodes isolated in quartz (ICAPPIC Limited, UK) with diameters 60–100 nm. All electrochemical measurements were carried out at room temperature using a two-electrode configuration with S4 silver chloride electrode as the counter-reference electrode. The Faradaic

current was measured with a A-M Systems 2000 patch-clamp amplifier. Transfer and recording of measurements to a computer was carried out using the ADC-DAC converter USB-6211 ("National instruments", USA) and the program WinWCP (John Dempster, University of Strathclyde, UK). The micromanipulator PatchStar (Scientifica, Great Britain) was used to feed the nanosensor. All manipulations were made on the table of an optical inverted microscope (Nikon, Japan). The current signals were filtered with 0.5 kHz lowpass filters, respectively. Cyclic voltammogram (CV) was measured in solution. The fabricated carbon nanoelectrodes were initially placed in 1 mM ferrocene methanol in PBS solution to verify their operability for further work. The initial electrode radius r was estimated from the steady-state current i_{ss} at 0.4 V in 1 mM ferrocene methanol in PBS according to the relation $i_{ss} = 4.64 \cdot r \cdot F \cdot c \cdot D$ with F the Faraday constant, c the concentration and D the diffusion coefficient (7.8×10^{-6} cm2 s-1 for FcMeOH). All carbon electrodes had a diameter between 30 and 100 nm. We then used the electrochemical method for creating cavities in carbon electrodes to ensure the optimal deposition of platinum. Electrochemical etching was performed by means of CV from 0 V to 2.2 V in 0.1 M KOH, 10 mM KCl for typically 15-40 cycles until the formation of cavities. At all stages of fabrication, we controlled the size of the electrodes. The electrochemical properties of the resulting electrodes are significantly different from disk electrodes. As a result of etching, two peaks appear on the voltammogram corresponding to the complete oxidation of FcMeOH and reduction of ferrocenium inside the nanocavity. Electrochemical deposition of platinum was achieved by cycling from 0 V to -0.8 V with a scan rate of 200 mV s-1 for 4-5 cycles in 2 mM H2PtCl6 solution in 0.1M hydrochloric acid.

Calibration of Pt nanoelectrodes

The total concentration of hydrogen peroxide was evaluated at a potential of +800 mV vs. Ag / AgCl. Under the given conditions, the platinum catalyzed reaction 2H2O2 = 2H2O + O2 occurs, and since the superoxide radical is rapidly converted in solution into hydrogen peroxide, the total concentration of peroxide determines the general background of the oxidation processes. Prior to the measurements, each platinum electrode was calibrated using a series of standard H2O2 solutions. Levels of ROS in the cells were determined based on the calibration curve. Deposition of a large amount of platinum leads to an increase in the sensitivity of the electrode, however, outgrowths are noticeable on the surface. They significantly damage the cell and are able to move away from the surface in a collision with the cell membrane. However, in our studies we used nanoelectrodes without outgrowths.
